# Enzymatically synthesized glycogen protects inflammation induced by urban particulate matter in normal human epidermal keratinocytes

**DOI:** 10.3164/jcbn.20-43

**Published:** 2020-06-05

**Authors:** Tomoya Kitakaze, Yasukiyo Yoshioka, Takashi Furuyashiki, Hitoshi Ashida

**Affiliations:** 1 Department of Agrobioscience, Graduate School of Agricultural Science, Kobe University, 1-1 Rokkodai-cho, Nada-ku, Kobe 657-8501, Japan; 2 Graduate School of Science, Technology and Innovation, Kobe University, 1-1 Rokkodai-cho, Nada-ku, Kobe 657-8501, Japan; 3 Institute of Health Sciences, Ezaki Glico Co., Ltd., 4-6-5 Utajima, Nishiyodogawa-ku, Osaka 555-8502, Japan

**Keywords:** enzymatically synthesized glycogen, particulate matter, inflammation, oxidative stress, normal human epidermal keratinocytes

## Abstract

Urban particulate matters (PM) exposure is significantly correlated with extrinsic skin aging signs and skin cancer incidence. PM contains polycyclic aromatic hydrocarbons, and they act as the agonists of aryl hydrocarbon receptor (AhR). Activation of AhR promotes generation of intracellular reactive oxygen species (ROS) and inflammation. Enzymatically synthesized glycogen (ESG), which is synthesized from starch, possesses various functions, such as anti-tumor, anti-obesity and antioxidant. However, the effects of ESG on PM-induced skin inflammation remain unclear. In this study, we investigated whether ESG has a protective effect on PM-induced oxidative stress and inflammation in human epidermal keratinocytes. ESG inhibited PM-induced expression of inflammatory cytokines *IL6*, *TNFA* and *PTGS2*. ESG also inhibited PM-induced phosphorylation of MAPKs and ROS accumulation. However, ESG had no effect on PM-induced expression of CYP1A1, one of the target proteins of AhR. On the other hand, ESG increased nuclear translocation of Nrf2 and expression of antioxidant proteins, HO-1 and NQO1. These results suggest that ESG suppressed PM-induced inflammation by decreasing ROS accumulation through the Nrf2 pathway.

## Introduction

Exposure to air pollution have a significant effect on human health. Exposure of urban particulate matters (PM), known as particulate matter less than 2.5 microns diameter (PM_2.5_) and that less than 10 microns diameter (PM_10_), has been linked to increased mortality, respiratory and cardiovascular morbidity, and reduced birth weight and increased post-neonatal mortality.^(1–4)^ Recent epidemiological studies have reported that urban PM exposure was significantly correlated with extrinsic skin aging signs and skin cancer incidence.^(5,6)^ Oxidative stress is the major cause of the PM-caused skin aging and diseases.^(7)^ A toxic substance of PM is well analyzed. In particular, several kinds of polycyclic aromatic hydrocarbons (PAHs) are adsorbed on the surface of suspended PM in air of urban areas, and PAHs can bind to the aryl hydrocarbon receptor (AhR), a ligand-activated transcription factor that mediates the toxicity of various xenobiotics, resulting in an increase of reactive oxygen species (ROS) generation and subsequent oxidative stress in the cells.^(8,9)^ PM-induced ROS generation activates nuclear factor (NF)-κB and mitogen-activated protein kinases (MAPKs), such as extracellular signal-regulated kinase 1/2 (ERK1/2), c-Jun N-terminal kinase (JNK), and p38 MAPK.^(10)^ Activation of these pathways leads to increase expression of pro-inflammatory cytokines, interleukin 6 (IL-6) and tumor necrosis factor-α (TNF-α), and inflammation related enzyme cyclooxygenase 2 (COX2). It has been reported that PM-caused oxidative stress promotes inflammatory response and leading to skin barrier dysfunction in human keratinocyte cells.^(11)^ Therefore, suppression of oxidative stress may provide a useful strategy for prevention of PM-caused skin disfunction.

Antioxidant enzymes play an important role in the protection against ROS-induced skin dysfunction. Nuclear factor-erythroid-2-related factor 2 (Nrf2) is a master transcriptional factor that regulates expression of antioxidant enzymes, including heme oxygenase 1 (HO-1) and NAD(P)H:quinone oxidoreductase 1 (NQO1).^(12)^ Induction of Nrf2 prevents UVB-induced cellular damage and depression of Nrf2 makes worse its cellular damage in a three-dimensional skin model.^(13)^ Thus, the activation of Nrf2 is a molecular target for prevention of skin dysfunction caused by oxidative stress.

Enzymatically synthesized glycogen (ESG) is a newly synthesized glycogen from plant starch.^(14)^ Although the physical properties of ESG are equivalent to those of natural sources of glycogens, its molecular structure is a slightly different from the structure of natural glycogens. Intake of ESG exerts certain biological effects such as immunostimulation activity, anti-diabetic effect, promotion of osteogenesis, inhibition of colitis and UVB-induced cell damage.^(15–20)^ Our previous study showed that an intake of ESG increases antioxidant enzymes through the activation of Nrf2 in macrophage of mice.^(19)^ However, it is unclear whether ESG is a useful skin care material for prevention of the toxicity caused by air pollution including PM. An aim of this study is to investigate the protective effect of ESG against PM-caused inflammation in human epidermal keratinocytes. Expression of proinflammatory cytokines and mediators and its upstream events were investigated to understand the inhibitory mechanism of ESG against PM-caused inflammatory response.

## Materials and Methods

### Materials

Enzymatically synthesized glycogen was prepared from plant starch as a substrate using three enzymes as previously described.^(18)^ The molecular weight of ESG is approximately 8,700 kDa.^(21)^ 2',7'-Dichlirodihydrofluorescein diacetate (DCFH-DA) was purchased from Sigma Aldrich (St. Louis, MO) For Western blotting, anti-CYP1A1 (Daiichi Pure Chemicals Co., Ltd., Tokyo, Japan), anti-NQO1 (Santa Cruz Biotechnology, Dallas, TX), anti-Nrf2 (Medical & Biologival Laboratories Co., Ltd., Aichi, Japan), anti-HO-1 (Enzo Life Sciences, Lausen, Switzerland), anti-ERK1/2 (Cell Signaling Technology, Danvers, MA), anti-p-ERK1/2 (Cell Signaling Technology), anti-p38 (Cell Signaling Technology), anti-p-p38 (Cell Signaling Technology), anti-JNK (Cell Signaling Technology), anti-p-JNK (Cell Signaling Technology), anti-lamin B (Santa Cruz Biotechnology), anti-IκBα (Cell Signaling Technology), and anti-β-actin (Cell Signaling Technology) antibodies were used in this study. Polyvinylidene difluoride membrane was a product of GE Healthcare Bio-Science Co. (Chicago, IL). All other reagents were of the highest grade available from commercial sources.

### Cell culture

The normal human epidermal keratinocytes (NHEK cells) were obtained from Kurabo Industries, Ltd. (Osaka, Japan) and cultured in keratinocyte growth medium (HuMedia-KG2; Kurabo Industries, Ltd.), containing insulin, hydrocortisone, gentamycin/amphotericin B and the growth additives including bovine pituitary extract and human epidermal growth factor under a humidified atmosphere of 95% (v/v) air and 5% (v/v) CO_2_ at 37°C.

### PM preparation

An environmental certified reference material 28 (CRM 28) is atmospheric particulate matter collected on filters in a central ventilating system in a building in Beijing city centre and was obtained from the National Institute for Environmental Studies (Ibaraki, Japan). The diameters of 99% of the particles are less than 10 µm and that of 40–60% of particles are less than 2.5 µm, and is containing PAHs. CMR 28 solution was prepared in dissolved in dimethyl sulfoxide (DMSO) and sonicated for 30 min to avoid agglomeration of the particles. CMR 28 solution was used in following experiments within 30 min after sonication as PM.

### Measurement of ROS accumulation

Intracellular ROS accumulation was monitored using a fluorogenic dye DCFH-DA according to the previous method.^(22)^ The cells were washed twice with PBS and incubated with PBS containing 10 µM DCFH-DA for 30 min. After washing twice with PBS, the cells were fixed by 4% paraformaldehyde for 20 min at 37°C. Then, cells were washed twice with PBS and treated with 1 µg/ml 4',6-diamidino-2-phenylindole (DAPI) for nuclear counter-staining. The fluorescence of DCF and DAPI were monitored at 485/535 nm and 355/460 nm, respectively, with an FSX100 microscope (Olympus, Tokyo, Japan). Separately, the fluorescence of DCF was quantified at 485/535 nm by a Wallac 1420 ARVOsx Multilabel Counter (Perkin-Elmer, Boston, MA).

### Cell fractionation

The cells were harvested and homogenized with RIPA buffer consisting of 20 mM Tris-HCl (pH 7.6), 150 mM NaCl, 0.5% (v/v) NP-40, 1 mM EDTA and 0.5 mM dithiothreitol containing protease inhibitors (1 mM phenylmethylsulfonyl fluoride, 5 µg/ml aprotinin and 5 µg/l leupeptin). Homogenate was centrifuged at 20,000 × *g* for 20 min at 4°C, and the resulting supernatant was referred to cell lysate. For preparing a nuclear fraction, after washing the cells twice with PBS, the cells were collected with lysis buffer consisting of 20 mM HEPES (pH 7.6), 20% glycerol, 10 mM NaCl, 1.5 mM MgCl_2_, 0.2 mM EDTA and 0.5 mM DTT containing the same protease and phosphatase inhibitors. The mixture was centrifuged at 800 × *g* for 10 min at 4°C, and the obtained precipitation was suspended in hypertonic buffer consisting of 20 mM HEPES (pH 7.6), 20% glycerol, 420 mM NaCl, 1.5 mM MgCl_2_, 0.2 mM EDTA and 0.5 mM DTT containing the same protease and phosphatase inhibitors. After left on ice for 1 h, the mixture was further centrifuged at 20,000 × *g* for 20 min at 4°C, and resultant supernatant was obtained and used as the nuclear fraction.

### Western blot analysis

Western blot analysis was performed as described previously.^(23)^ The cell lysate and nuclear fraction were subjected to SDS-PAGE, followed by Western blot analysis using following antibodies: anti-CYP1A1 (1:10,000), anti-NQO1 (1:5,000), anti-HO-1 (1:20,000), anti-Nrf2 (1:5,000), anti-ERK1/2 (1:5,000), anti-p-ERK1/2 (1:5,000), anti-p38 (1:5,000), anti-p-p38 (1:5,000), anti-JNK (1:5,000), anti-p-JNK (1:5,000), anti-lamin B (1:5,000), anti- IκBα (1:5,000) and β-actin (1:5,000) overnight at 4°C, followed by incubation with the corresponding HRP-conjugated secondary antibody (1:20,000–1:50,000) for 1 h at room temperature. The blot was developed using Immuno Star LD Western Blotting Substrate (FUJIFILM Wako Pure Chemical Corporation, Osaka, Japan) and immunocomplexes were detected with Light-Capture II (ATTO Co., Tokyo, Japan). The density of the specific band was determined using ImageJ image analysis software ver. 1.44 (National Institutes of Health, Bethesda, MD).

### RNA isolation and real-time PCR analysis

The total RNA from the cells was isolated using TRIzol (Thermo Fisher Scientific, Waltham, MA) in accordance with the manufacturer’s instruction, and subjected to the reverse transcriptional reaction. The resultant cDNA was subjected to a real-time PCR system (TAKARA PCR Thermal Cycler Dice, Takara Bio, Shiga, Japan) using SYBR Premix Ex Taq II (Takara Bio). Following specific primers were used: *PTGS2* (forward primer 5'-CCTTGGGTG TCAAAGGTAA-3' and reverse primer 5'-GCCCTCGCTTAT GATCTGTC-3'); *TNFA* (forward primer 5'-GGAGAAGGG TGACCGACTCA-3' and reverse primer 5'-TGCCCAGACTCG GCAAAG-3'); *IL6* (forward primer 5'-GGAGACTTGCCTGGT GAAAA-3' and reverse primer 5'-GTCAGGGGTGGTTAT TGCAT-3'); and *ACTB* (forward primer 5'-GGACTTCGA GCAAGAGATGG-3' and reverse primer 5'-AGCACTGTGTTG GCGTACAG-3'). *ACTB* mRNA was used as a normalized control.

### Statistical analysis

Data are expressed as the mean ± SD of at least three independent determinations for each experiment. Dunnett’s test or Tukey Kramer multiple comparison test was used to determine the significant difference among the experimental groups. The Student’s *t* test was also used for determining significant differences between two experimental groups. The level of statistical significance was set as *p*<0.05.

## Results

### Effect of ESG on PM-induced inflammation in NHEK cells

**Fig. 1:**
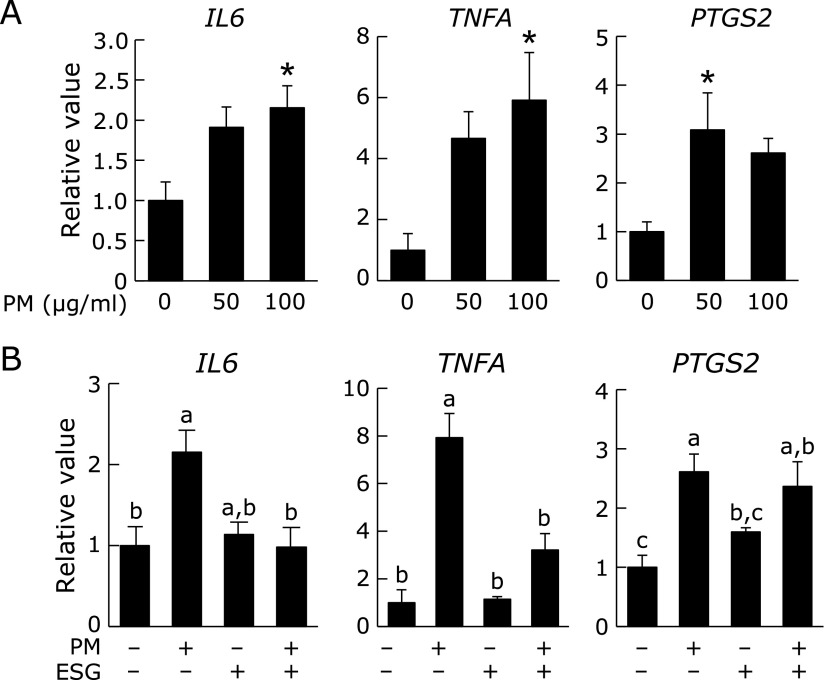
ESG inhibited PM-induced inflammation in NHEK cells. (A) NHEK cells were treated with 0–100 µg/ml PM or DMSO as a vehicle control for 24 h. (B) NHEK cells were pre-treated with 600 µg/ml ESG or PBS as a vehicle control for 24 h. The cells were incubated with 0–100 µg/ml PM or DMSO as a vehicle control for another 24 h. The mRNA expression level of *IL6*, *TNFA* and *PTGS2* was measured by real-time PCR. ACTB was used as a control. Data were normalized to the *ACTB* mRNA level. The results are represented as the mean ± SD (*n* = 3–5). (A) Asterisks indicate a significant difference from the value of vehicle-treated cells by Dunnett’s test (*p*<0.05). (B) Means with different letters differ significantly (*p*<0.05), as determined by the Tukey-Kramer test.

To investigate the PM-induced inflammation, expression of inflammatory cytokines was evaluated. Expression of *IL6*, *TNFA* and *PTGS2* was dose-dependently increased by treatment with 0–100 µg/ml PM (Fig. 1A), indicating that PM induced inflammation in NHEK cells as expected. When the cells were pre-treated with ESG, PM-induced upregulation of *IL6*, *TNFA* and *PTGS2* was significantly suppressed (Fig. 1B).

### Effect of ESG on PM-activated MAPK signaling pathways in NHEK cells

**Fig. 2:**
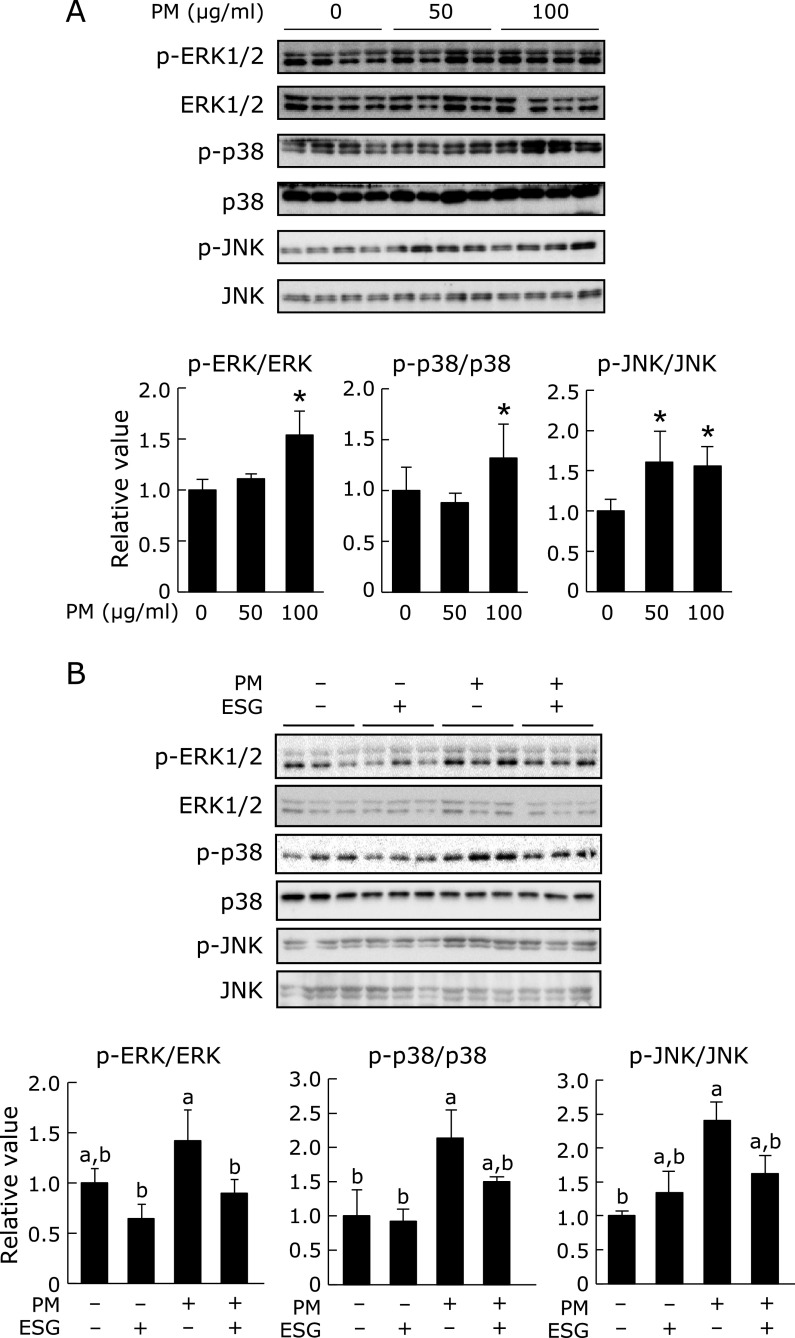
ESG inhibited PM-induced phosphorylation of MAPKs in NHEK cells. (A) NHEK cells were treated with 0–100 µg/ml PM or DMSO as a vehicle control for 24 h. (B) NHEK cells were pre-treated with 600 µg/ml ESG or PBS as a vehicle control for 24 h. The cells were incubated with 0–100 µg/ml PM or DMSO as a vehicle control for another 24 h. The protein level of p-ERK1/2, ERK1/2, p-p38, p38, p-JNK and JNK was determined by Western blot analysis with their respective antibodies (top panels). The intensity of each specific band was quantified by ImageJ 1.44 (bottom panels). The results are represented as the mean ± SD (*n* = 3). (A) Asterisks indicate a significant difference from the value of vehicle-treated cells by Dunnett’s test (*p*<0.05). (B) Means with different letters differ significantly (*p*<0.05), as determined by the Tukey-Kramer test.

**Fig. 3:**
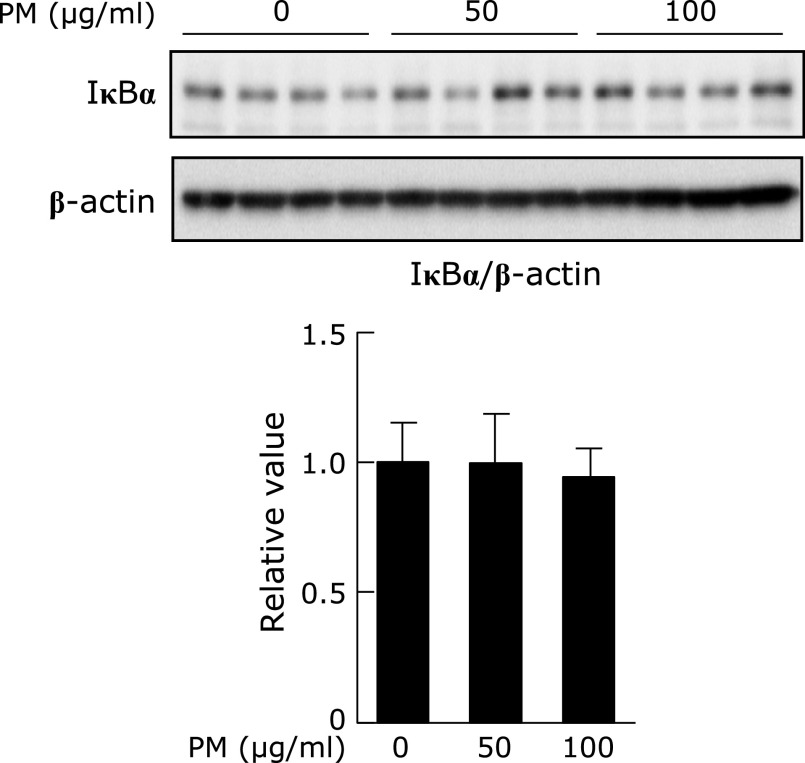
PM had no influence on expression of IκBα in NHEK cells. NHEK cells were treated with 0–100 µg/ml PM or DMSO as a vehicle control for 24 h. The protein level of IκBα and β-actin was determined by Western blot analysis with their respective antibodies (top panels). The intensity of each specific band was quantified by ImageJ 1.44 (bottom panels). The results are represented as the mean ± SD (*n* = 3).

It has been reported that MAPK signaling pathway mediated PM-induced inflammation in HaCaT cells.^(11)^ As shown in Fig. 2A, 0–100 µg/ml PM increased phosphorylation of ERK1/2, p38 and JNK dose-dependently in NHEK cells. When the cells were pre-treated with ESG, PM-induced phosphorylation of these MAPKs was significantly suppressed (Fig. 2B). To evaluate the effect of PM on the NF-κB signaling pathway, expression of NF-κB inhibitor α (IκBα), a negative regulator of NF-κB signaling pathway, was determined. As a result, PM had no effect on expression of IκBα (Fig. 3).

### Effect of ESG on PM-induced ROS accumulation in NHEK cells

**Fig. 4:**
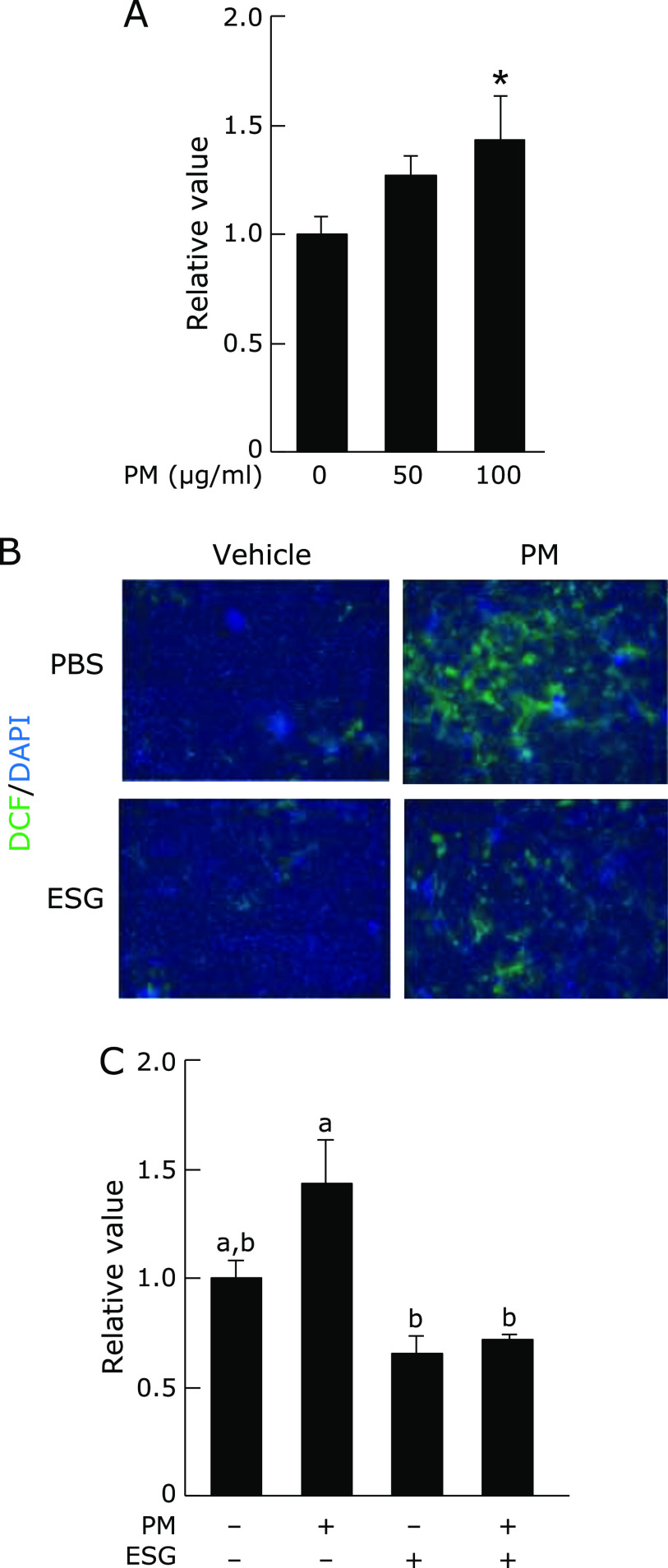
ESG inhibited PM-induced ROS accumulation in NHEK cells. (A) NHEK cells were treated with 0–100 µg/ml PM or DMSO as a vehicle control for 24 h (B and C). NHEK cells were pre-treated with 600 µg/ml ESG or PBS as a vehicle control for 24 h. The cells were incubated with 0–100 µg/ml PM or DMSO as a vehicle control for another 24 h, and then incubated with 10 µM DCFH-DA in PBS for another 30 min. (A and C) The fluorescent intensity of DCF was measured at 485/535 nm. (B) After treatment with DCFH-DA, the cells were fixed for 20 min and treated with 1 µg/ml DAPI. The fluorescence of DCF (red) and DAPI (blue) was visualized at 485/535 nm and 355/460 nm, respectively. The results are represented as the mean ± SD (*n* = 3). (A) Asterisks indicate a significant difference from the value of vehicle-treated cells by Dunnett’s test (*p*<0.05). (C) Means with different letters differ significantly (*p*<0.05), as determined by the Tukey-Kramer test.

To evaluate the PM-induced ROS accumulation, DCFH-DA was introduced. As shown in Fig. 4A, treatment with 0–100 µg/ml PM increased ROS accumulation after 24 h dose dependently. When the cells were pre-treated with ESG, PM-induced ROS accumulation was significantly suppressed (Fig. 4B and C).

### Effect of ESG on PM-activated AhR in NHEK cells

**Fig. 5:**
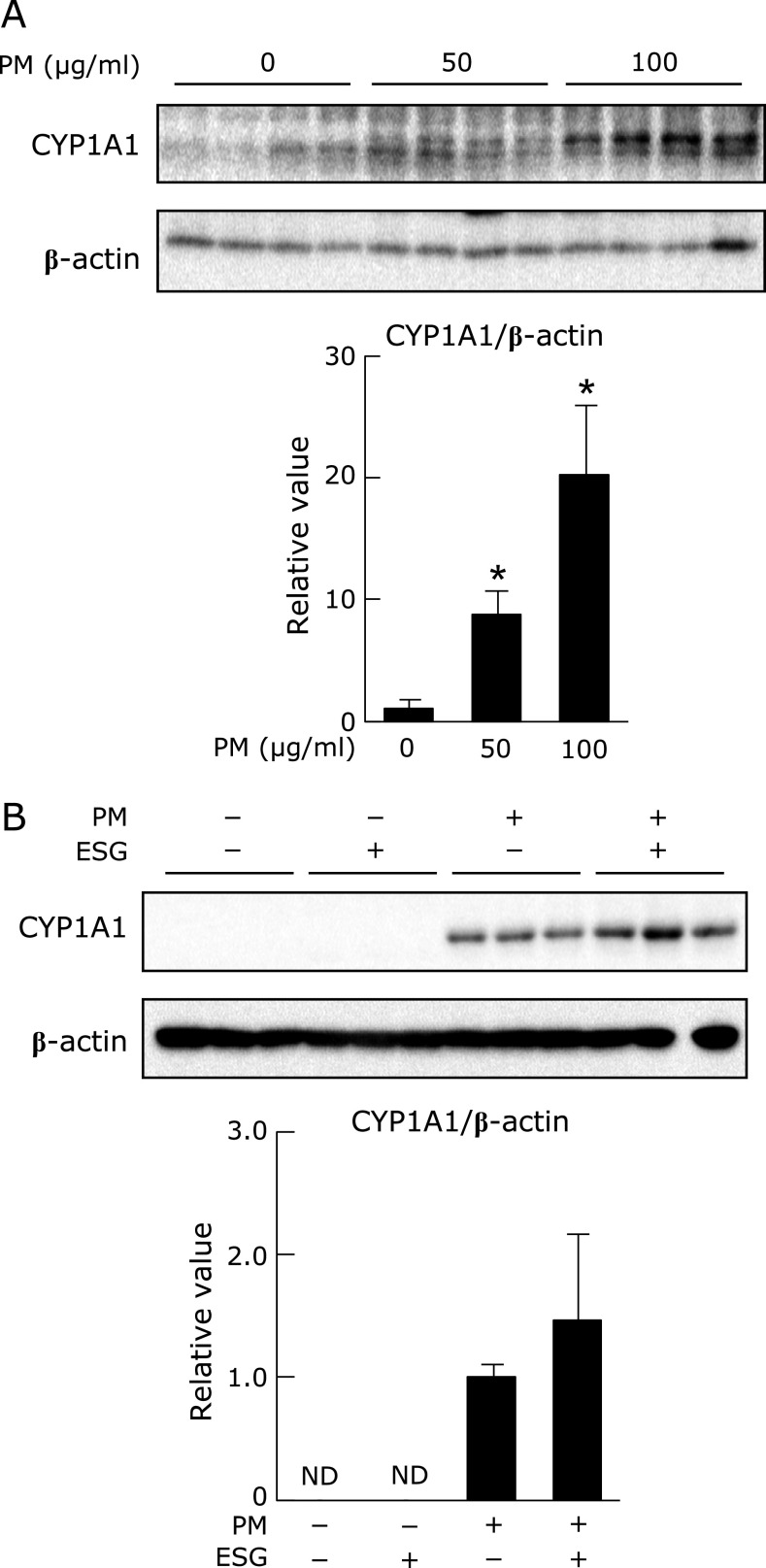
ESG had no effect on PM-induced expression of CYP1A1 in NHEK cells. (A) NHEK cells were treated with 0–100 µg/ml PM or DMSO as a vehicle control for 24 h. (B) NHEK cells were pre-treated with 600 µg/ml ESG or PBS as a vehicle control for 24 h. Then, cells were incubated with 0–100 µg/ml PM or DMSO as a vehicle control for another 24 h. The protein level of CYP1A1 and β-actin was determined by Western blot analysis with their respective antibodies (top panels). The intensity of each specific band was quantified by ImageJ 1.44 (bottom panels). The results are represented as the mean ± SD (*n* = 3). Asterisks indicate a significant difference from the value of vehicle-treated cells by Dunnett’s test (*p*<0.05).

 The activation of AhR results in increased ROS generation.^(11)^ Therefore, we next investigated the effect of PM and ESG on the activation of AhR. To evaluate the activation of AhR, expression of its target protein, cytochrome P450 1A1 (CYP1A1) was measured. PM dramatically increased expression of CYP1A1 dose-dependently in NHEK cells (Fig. 5A). Pre-treatment with ESG had no effect on PM-induced expression of CYP1A1 (Fig. 5B). These results suggested that ESG suppresses PM-induced ROS accumulation through indirectly pathway but not directly inhibiting the AhR pathway in NHEK cells.

### Effect of ESG on Nrf2 pathway in NHEK cells

**Fig. 6:**
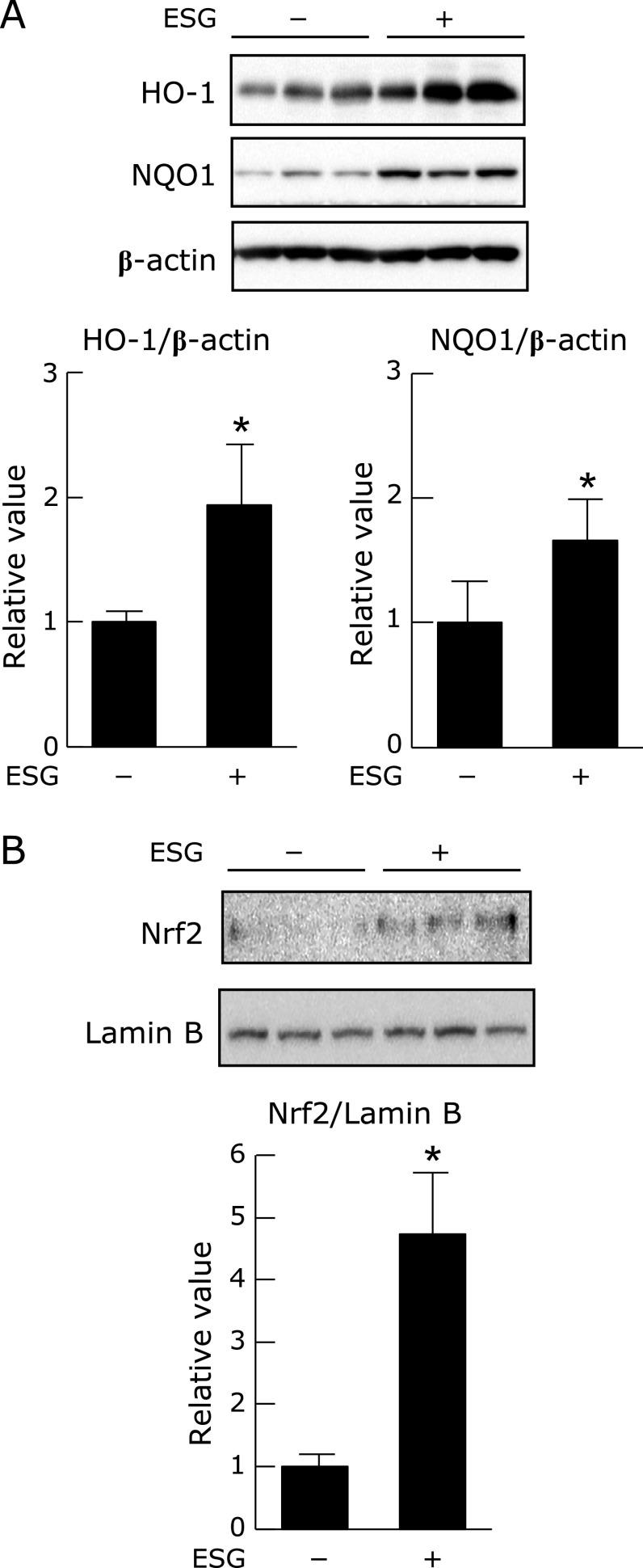
ESG increased nuclear accumulation of Nrf2 and expression of antioxidant proteins in NHEK cells. (A) NHEK cells were treated with 600 µg/ml ESG or PBS as a vehicle control for 24 h. The protein levels of HO-1, NQO1 and β-actin were determined by Western blot analysis with their respective antibodies (top panels). The intensity of each specific band was quantified by ImageJ 1.44 (bottom panels). (B) NHEK cells were treated with 600 µg/ml ESG or PBS as a vehicle control for 1 h. Expression level of nuclear Nrf2 and lamin B was determined by Western blot analysis with their respective antibodies (top panels). The intensity of each band was quantified by ImageJ 1.44 (bottom panels). The results are represented as the mean ± SD (*n* = 3). Asterisks indicate a significant difference from the value of the vehicle-treated cells by the Student *t* test (*p*<0.05).

Since our previous study demonstrated that ESG activates Nrf2 pathway and increases its target antioxidant proteins, HO-1 and NQO1 in murine macrophages,^(19)^ expression of antioxidant proteins and nuclear translocation of Nrf2 were evaluated in NHEK cells. ESG significantly increased expression of HO-1 and NQO-1 in NHEK cells (Fig. 6A). Nuclear translocation of Nrf2 was also increased in NHEK cells 1 h after the ESG treatment (Fig. 6B). These results indicated that ESG protects PM-induced ROS accumulation by the induction of antioxidant proteins through the Nfr2 pathway in NHEK cells.

## Discussion

In this study, the authors demonstrated that ESG prevented PM-induced oxidative stress and inflammation in NHEK cells. PM exposure activated AhR and increased ROS generation, and following activation of MAPK signaling pathway and inflammatory response in NHEK cells. Pre-treatment with ESG to NHEK cells inhibited PM-induced phosphorylation of MAPKs and ROS generation but not expression of CYP1A1. On the other hand, ESG increased nuclear translocation of Nrf2 and expression of antioxidant enzymes. These results suggest that ESG suppressed PM-induced inflammation by decreasing the ROS accumulation through the Nrf2 pathway but not inhibiting the AhR pathway. This is a first report that ESG attenuated PM-induced inflammation by modulating the Nrf2 pathway.

The skin is directly exposed to the air pollutant and skin diseases is induced by airborne PM. It has been known that PM-induced skin diseases and skin aging are largely mediated by ROS,^(7)^ and the harmful effects of PM may be ameliorated by antioxidant compounds. Recent studies have reported that several phenolic compounds derived from edible plants, such as (−)-epigallocatechin gallate and resveratrol, attenuated cellular oxidative stress induced by PM.^(24,25)^ These phenolic compounds possess antioxidant activity not only by exerting free-radical scavenging property directly but also by activating antioxidant enzymes through the Nrf2 pathway indirectly. In this study we firstly demonstrated that non phenolic compound glycogen can be useful for skin health against PM-induced oxidative stress and inflammation through activating the Nrf2 pathway.

It is still unclear that the underlying mechanism by which ESG induced Nrf2 activation, but several studies reported that ESG interacts with the proteins localized in plasma membrane, Toll-like receptor 2 (TLR2) and glucose transporter 1 (GLUT1).^(17,26)^ In macrophage, ESG enhanced the NF-κB activity though TLR2. Peptidoglycan, a TLR2 ligand, increased expression of HO-1 by promoting the degradation of Kelch-like ECH-associated protein 1 which is a negative regulator of Nrf2.^(27)^ Furthermore, it has been reported that overexpression of TLR2 increases expression of HO-1 and Nrf2 in dairy goat macrophage.^(28)^ On the other hand, ESG-induced phosphorylation of Akt was inhibited by phloretin, a GLUT1 inhibitor.^(17)^ Akt is known to induce phosphorylation of Nrf2 at Ser40,^(29,30)^ resulting in increased Nrf2 nuclear translocation.^(23)^ These results suggest that both TLR2 and GLUT1 are candidates of ESG target proteins which are upstream of ESG-induced Nrf2 activation. Meanwhile, we previously demonstrated that resistant glycogen, a digestion product of ESG by α-amylase, induces phosphorylation of Nrf2 at Ser40 through the ERK1/2 and JNK signaling pathways in mice macrophage.^(19)^ Further studies are needed to clarify the upstream event of ESG-induced Nrf2 activation in future.

In this study we showed that ESG inhibited PM-induced inflammation by increasing antioxidant capacity in NHEK cells. PM-induced activation of AhR causes skin aging, skin cancer and atopic dermatitis.^(31)^ PM-increased expression of COX2 (encoded by the *PTGS2* gene) induced production of prostaglandins and activated following prostaglandin receptor-mediated downregulation of filaggrin. Filaggrin plays a key role in conferring keratinocytes with their physical strength via aggregation of keratin bundles and contributes to epidermal hydration and barrier function.^(11)^ PM-induced inflammatory response is reported to depend on the AhR signaling.^(11)^ On the other hand, it has been reported that the binding of PM_2.5_ to TLR5 initiated the intracellular signaling through MyD88, and led to the activation of NF-κB signaling.^(32)^ Furthermore, PM_2.5_ induced direct interaction between TLR5 and NADPH oxidase 4, and subsequently increased the production of ROS and activated downstream NF-κB signaling. These results suggest that inhibition of ROS accumulation contributed to the protection of PM-induced inflammation mediated by both AhR and TLR5 pathways. The results from these previous reports supported our finding that ESG inhibited PM-induced expression of *IL6*, *TNFA* and *PTGS2*, although PM had no effect on expression of IκBα in this study.

**Fig. 7:**
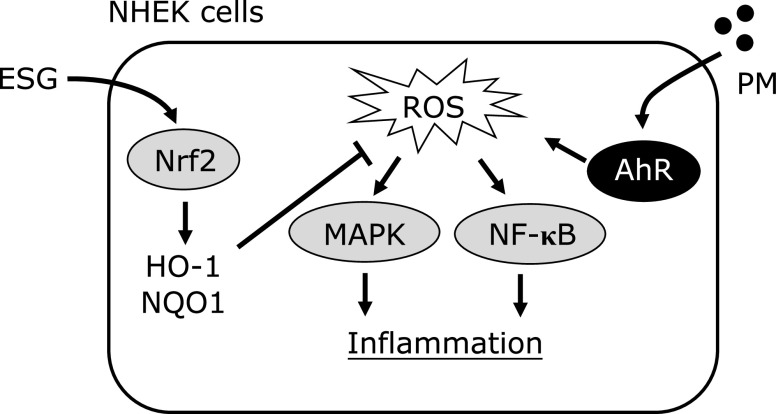
Putative molecular mechanism of ESG for prevention of PM-induced inflammation in NHEK cells.

In conclusion, ESG suppressed PM-induced ROS accumulation and inflammation in NHEK cells. As a putative mechanism, ESG suppressed PM-induced inflammation by decreasing the ROS accumulation through the Nrf2 pathway (Fig. 7). Our findings suggest that ESG is an effective compound for prevention of PM-induced skin diseases. Therefore, ESG is a useful material for the skin care products.

## Abbreviations

AhR: aryl hydrocarbon receptor
COX2: cyclooxygenase 2
CRM 28: certified reference material 28
CYP1A1: cytochrome P450 1A1
DAPI: 4',6-diamidino-2-phenylindole
DCFH-DA: 2',7'-dichlirodihydrofluorescein diacetate
DMSO: dimethyl sulfoxide
ERK1/2: extracellular signal-regulated kinase 1/2
ESG: enzymatically synthesized glycogen
GLUT1: glucose transporter 1
HO-1: heme oxygenase 1
IκBα: NF-κB inhibitor α
IL-6: interleukin 6
JNK: c-Jun N-terminal kinase
MAPK: mitogen-activated protein kinase
NF-κB: nuclear factor-κB
NQO1: NAD(P)H:quinone oxidoreductase 1
Nrf2: nuclear factor-erythroid-2-related factor 2
PAH: polycyclic aromatic hydrocarbons
PM: particulate matters
ROS: reactive oxygen species
TLR2: Toll-like receptor 2
TNF-α: tumor necrosis factor-α
